# Comparative Analysis of Chemiluminescence Immunoassay (CLIA)‐Based Tests in the Diagnosis of Invasive Aspergillosis in Patients With Hematologic Malignancies

**DOI:** 10.1111/myc.70064

**Published:** 2025-04-25

**Authors:** Esra Kılıç, Elif Ayça Şahin, Özlem Güzel Tunçcan, Şeyma Yıldız, Zübeyde Nur Özkurt, Zeynep Arzu Yeğin, Ayşe Kalkancı

**Affiliations:** ^1^ Department of Medical Microbiology Gazi University Faculty of Medicine Ankara Turkiye; ^2^ Department of Infectious Diseases and Clinical Microbiology Gazi University Faculty of Medicine Ankara Turkiye; ^3^ Department of Hematology Gazi University Faculty of Medicine Ankara Türkiye

**Keywords:** (1–3) beta‐D‐glucan, aspergillosis, chemiluminescence immunoassays (CLIA), Dynamiker ELISA, galactomannan, Platelia *Aspergillus* ELISA

## Abstract

**Background and Aim:**

Rapid chemiluminescence immunoassays (CLIA) have emerged as a promising alternative to traditional serological methods for the diagnosis of invasive aspergillosis (IA). The aim of this study was to compare the diagnostic performance of rapid CLIA tests in IA.

**Methods:**

Patient group consisted of 17 patients who were diagnosed with probable IA according to EORTC/MSG criteria. Patients without invasive fungal infection (IFI) were defined as the control group, whereas healthy volunteers were also included. A total of 93 serum samples were used in this study. Platelia *Aspergillus* Ag test and Dynamiker *Aspergillus* Ag Kit, CLIA tests *Aspergillus* Galactomannan Detection Kit and Fungus (1–3) ꞵ‐D‐Glucan Detection Kit, were used. Specificity, sensitivity, negative predictive value (NPV), and positive predictive value (PPV) were calculated. Receiver operating characteristic (ROC) curve was used to evaluate the overall diagnostic performance of CLIA tests comparing FDA‐approved GM ELISA test.

**Results:**

The sensitivity of the CLIA galactomannan (CLIA GM) test was 70.6%, specificity 92.1%, PPV 66.7% and NPV 93.3% (*p* < 0.001), while the sensitivity of the CLIA beta‐glucan (CLIA BDG) test was 88.2%, specificity 81.6%, PPV 51.7% and NPV 96.9% (*p* < 0.001). Using the PlateliaTM *Aspergillus* Ag Test as the reference method, the areas under the curve (AUC) of the ROC curve were 0.878 for CLIA BDG and 0.869 for CLIA GM.

**Conclusions:**

CLIA‐based tests were evaluated as being rapid diagnostic tests for IA since their NPVs were found to be very high. Integrating CLIA into clinical practice may significantly improve diagnostic efficiency and patient outcomes.

## 1 Introduction

Invasive fungal infections (IFIs), unlike superficial fungal infections, are severe systemic infections that affect deep tissues and cause high mortality rates, particularly in immunocompromised patients. These infections are common in patient populations with hematologic malignancies, chemotherapy‐induced neutropenia, haematopoietic stem cell transplantation (HSCT), and solid organ transplantation [1, 2]. Pathogens such as *Candida*, *Aspergillus*, *Cryptococcus* and *Pneumocystis* species frequently cause these infections. Diagnosing IFIs involves microbiological examination of blood or tissue samples. However, obtaining samples through invasive methods often delays diagnosis and limits early intervention. Rapid, noninvasive, culture‐independent diagnostic methods are essential for timely antifungal treatment. Among these methods, serological diagnostic tests focus on detecting biomarkers such as galactomannan (GM) from *Aspergillus* species and mannan antigens from *Candida* species in serum. Additionally, (1–3)‐β‐D‐glucan (BDG), a biomarker found in most fungi, is widely used in IFI diagnosis [3, 4, 5, 6, 7]. The European Organization for Research and Treatment of Cancer and the Mycoses Study Group (EORTC/MSG) criteria are commonly employed to classify infections into proven, probable and possible categories. These criteria particularly utilise serum GM antigen detection to diagnose probable invasive aspergillosis (IA) in culture‐negative cases [3, 8].

Among serological tests for aspergillosis diagnosis, ELISA is the most widely used method. Although highly sensitive, obtaining results from a single sample can be challenging [9, 10]. Recently developed fully automated chemiluminescence immunoassay (CLIA) systems offer an alternative for rapid diagnosis [11, 12]. This study aimed to compare the performance of two commercial CLIA‐based tests, the *Aspergillus* Galactomannan (GM CLIA) Detection Kit and the Fungus (1–3)‐β‐D‐Glucan (BDG CLIA) Detection Kit, against the FDA‐approved Platelia *Aspergillus* Ag ELISA test and another second galactomannan ELISA (Dynamiker Aspergillus Galactomannan Assay).

## 2 Materials and Methods

### 2.1 Study Design and Patients

The study was conducted after receiving approval from the Gazi University Faculty of Medicine Clinical Research Ethics Committee with decision number 658 in the meeting dated 31 July 2023 and from the Turkish Medicines and Medical Devices Agency of the Republic of Turkey Ministry of Health with the decision dated 22.08.2023 and numbered E‐68869993‐000‐1199247. The study included a total of 93 individuals: 17 patients with probable IA, 49 patients without IFI suspicion and 27 healthy volunteers between October 2023 and June 2024. Patient age, gender, underlying diseases, presence of HSCT, development of graft versus host disease (GVHD), use of immunosuppressive drugs and whether prophylactic mould‐active antifungal treatment was received were evaluated as demographic data. Serum samples were stored at −80°C in a New Brunswick Scientific deep freezer.

### 2.2 ELISA Testing

Two ELISA tests, Platelia *Aspergillus* (GM ELISA, Bio‐Rad, USA) and Dynamiker *Aspergillus* Galactomannan Assay (Dynamiker (DymGM, ELISA, Tianjin) Co. Ltd., China), were used to detect GM antigens in serum samples. These studies were conducted with a 300‐μl samples. GM antigens bind to monoclonal antibodies in microwell plates, and the reaction was detected spectrophotometrically at 450 nm. For both ELISA tests, the average cut‐off control value was determined by dividing the total serum optical densities of the cut‐off control by two. An index ≥ 0.5 was considered a positive result.

### 2.3 CLIA Method

Chemiluminescence‐based GM and BDG analyses were performed using the automated chemiluminescence immunoassay device (FACIS‐1, Genobio, China) and using appropriate kits. Fungus (1‐3)‐β‐D‐Glucan Detection Kit (BDG CLIA) for BDG and Aspergillus Galactomannan Detection Kit (GM CLIA) for GM were used (Genobio, China). The CLIA method detects antigen–antibody reactions using luminol‐based substrates. Reactions were measured via emitted light intensity to calculate antigen concentrations. A total of 300 μL of sample/control were added and pipetted, and the test was initiated. A calibration curve was generated using calibration data recorded by the system. The system software automatically calculated the antigen concentration of each sample using a weighted four‐parameter fitting method. A result in ng/mL units was obtained in approximately 1 h. To ensure the reliability of the test results, each kit batch was tested at least twice, and the results were found to be within the specified control ranges. For GM CLIA, the reference range for the positive control was 8–12 μg/L, and the reference range for the negative control was 0–0.25 μg/L. A GM concentration of ≥ 0.5 μg/L was considered a positive result. A GM concentration of < 0.25 μg/L was considered a negative result. GM concentrations between 0.25 and 0.5 μg/L were regarded as indeterminate results. The reference range of BDG CLIA for the positive control was 8–12 ng/mL, while the reference range for the negative control was 0–0.06 ng/mL. A BDG concentration of ≥ 0.1 ng/mL was considered a positive result. A BDG concentration of < 0.06 ng/mL was considered a negative result. BDG concentrations between 0.06 and 0.1 ng/mL were regarded as indeterminate results.

### 2.4 Statistical Analysis

The statistical analyses of the research data were performed using the Statistical Package for Social Sciences (SPSS), version 25.0 for Windows (SPSS Inc., Chicago, USA). In the descriptive statistics section, categorical variables were presented as numbers and percentages, while continuous variables were presented as mean ± standard deviation and median (minimum–maximum values). For the GM and BDG tests, sensitivity, specificity, positive predictive value (PPV) and negative predictive value (NPV) were calculated. To evaluate the compatibility between tests, Receiver Operating Characteristic (ROC) analysis was performed, and the area under the curve (AUC) was calculated. The AUC was interpreted as follows: 0.90–1.00 excellent, 0.80–0.90 good, 0.70–0.80 moderate, 0.60–0.70 poor, and 0.50–0.60 failed. A *p*‐value of < 0.05 was considered statistically significant.

The correlation between Platelia GM ELISA, DynGM ELISA, GM CLIA and BDG CLIA was analysed using Spearman's rank correlation coefficient (Spearman's rho). This nonparametric statistical test was employed to assess the strength and direction of monotonic relationships between the variables. Given the nature of the data, Spearman's rho was chosen as it does not assume a normal distribution. The correlation coefficients were calculated along with their corresponding two‐tailed significance (*p* values). A significance threshold of 0.01 (*p* < 0.01) was applied to determine statistically significant associations. The sample size (*N*) for each correlation was 93 observations.

## 3 Results

### 3.1 Demographic Data

According to the EORTC/MSG criteria [3], the group defined as having a probable IA consisted of 7 (41.18%) female and 10 (58.82%) male patients, with a mean age of 50.00 ± 16.82 years. The probable IA group consisted of GM ELISA positive cases. The control group without IFI included 23 (46.9%) female and 26 (53.1%) male patients, with a mean age of 51 ± 13.4 years. In the healthy control group, there were 15 (55.6%) female and 12 (44.4%) male participants, with a mean age of 50.44 ± 17.04 years. In the patient group with a probable IA, the most common underlying disease was acute myeloid leukaemia (AML) (52.94%), followed by lymphoma (23.53%) and acute lymphoblastic leukaemia (ALL) (17.65%). In the control group without IFI, the most common underlying disease was AML (46.94%), followed by ALL (16.33%) and lymphoma (14.29%).

### 3.2 Test Results and Diagnostic Accuracy

In our study, a total of 93 patient serum samples were tested. The FDA‐approved Platelia *Aspergillus* Ag test was accepted as the reference serological method. Table 1 shows comparative results of four serological methods. The DynGM ELISA was found to be positive in 17 GM ELISA‐positive samples, whereas two additional negative samples were found to be false‐positive by DynGM ELISA. These two patients had no IFIs; one was AML, and the second one was a multiple myeloma patient (Table 1). A total of 19 samples were found to be DynGM ELISA positive. The Dynamiker *Aspergillus* GM test demonstrated a sensitivity of 100%, specificity of 97.4%, PPV of 89.5% and NPV of 100% based on a single serum sample. ROC analysis was performed using the Platelia GM test as the reference method. According to the AUC values of the ROC curve, the DynGM ELISA test was found to be 99.4% accurate (0.994) demonstrating excellent diagnostic performance.

**TABLE 1:** Comparative analysis of serological results.

		Probable IA (17)	Control group without IFIs (49)	Healthy control (27)	Total (93)
Platelia GM ELISA	Positive	17	0	0	17
	Negative	0	49	27	76
*Reference test*					
Dynamiker GM ELISA	Positive	17	2	0	19
	Negative	0	47	27	74
*Sensitivity 100%, specificity 97.4%, PPV 89.5%, NPV 100%, accuracy 99.4%*					
GM CLIA	Positive	12	2	4	18
	Negative	5 (Fungal pneumonia)	47	23	75
*Sensitivity 70.6%, specificity 92.1%, PPV 66.7%, NPV 93.3%, accuracy 86.9%*					
BDG CLIA	Positive	15	8	6	29
	Negative	2 (Fungal pneumonia)	41	21	64
*Sensitivity 88.2%, specificity 81.6%, PPV 51.7%, NPV 96.9%, accuracy 87.8%*					

GM CLIA test was found to detect positivity in 12 of the 17 samples identified as positive by the GM ELISA while it yielded negative results in five of these samples. These five patients were classified as probable IA presenting signs of fungal pneumonia (Table 1). Additionally, the GM CLIA test detected positivity in six samples that were negative according to the GM ELISA test. Overall, the GM CLIA test identified positivity in a total of 18 samples. According to the results of the GM ELISA test, the GM CLIA test demonstrated a sensitivity of 70.6%, specificity of 92.1%, PPV of 66.7%, and NPV of 93.3%. ROC analysis was performed for the GM CLIA test using GM ELISA as the reference standard. According to the AUC value of the ROC curve, the GM CLIA test was found to be 86.9% accurate.

**FIGURE 1:**
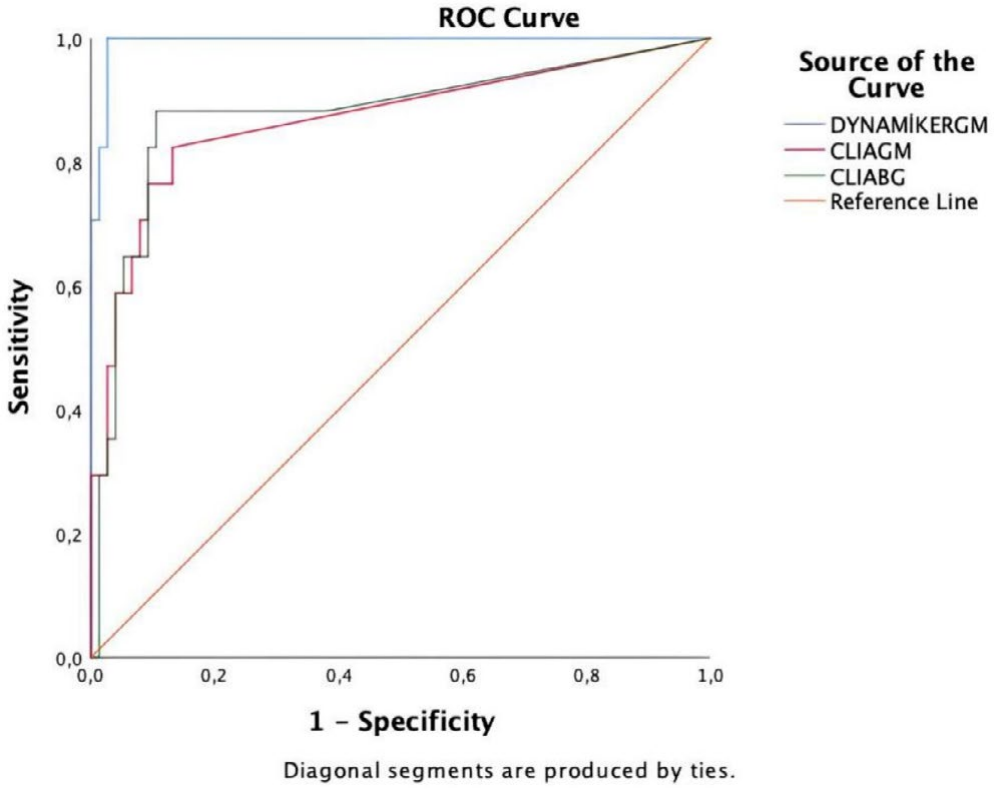
The ROC analysis was performed for the Dynamiker GM, GM CLIA and BDG CLIA tests using the Platelia GM test as the reference.

BDG CLIA test detected positivity in 15 of the 17 samples identified as positive by GM ELISA test, while it yielded negative results in two of these samples. Two additional GM ELISA‐negative samples were found to be false‐positive by DynGM ELISA. These two patients, one with AML and the other with multiple myeloma, were from the control group and did not fulfil the EORTC/MSG criteria for probable or possible IFI (Table 1). Additionally, the BDG CLIA test was found to be positive in 14 samples that were negative according to the GM ELISA test. Overall, the BDG CLIA test identified positivity in a total of 29 samples. According to the results of the FDA‐approved GM ELISA test, the BDG CLIA test demonstrated a sensitivity of 88.2%, specificity of 81.6%, PPV of 51.7% and NPV of 96.9% based on a single serum sample. ROC analysis was performed for the BDG CLIA test using the GM ELISA test as the reference standard. According to the AUC value of the ROC curve, the BDG CLIA test was found to be 87.8% accurate. Figure 1 and Table 2 show ROC analysis.

**TABLE 2:** Area under curve—AUC and data used for ROC analysis.

	AUC	Standard deviation	*p*	95% Confidence interval	
				Lower bound	Upper bound
Dynamiker GM ELISA	0.994	0.005	0.000	0.983	1.000
GM CLIA	0.869	0.058	0.000	0.755	0.983
BDG CLIA	0.878	0.056	0.000	0.768	0.989

A combined ROC analysis was performed for the four tests. According to the AUC values of the ROC curve, the ranking of performance based on the reference method was as follows: DynGM ELISA 0.994, BDG CLIA 0.878 and GM CLIA 0.869.

Spearman's rank correlation analysis revealed statistically significant monotonic relationships between Platelia GM ELISA, DynGM ELISA, GM CLIA and BDG CLIA (*p* < 0.01 for all correlations). The highest correlation was observed between Platelia GM ELISA and DynGM ELISA (*ρ* = 0.618, *p* < 0.01), indicating a moderate‐to‐strong positive association. GM CLIA demonstrated weaker correlations with both Platelia GM ELISA (*ρ* = 0.352, *p* < 0.01) and Dynamiker GM ELISA (*ρ* = 0.364, *p* < 0.01), suggesting a moderate relationship. Additionally, BDG CLIA showed moderate correlations with all other assays, with the highest value observed between BDG CLIA and Dynamiker GM ELISA (*ρ* = 0.483, *p* < 0.01). These findings suggest that while the assays share common trends, their rankings exhibit varying degrees of agreement. Table 3 shows the results of this analysis.

**TABLE 3:** Spearman's rank correlation coefficients (rho) between Platelia GM ELISA, Dynamiker GM ELISA, GM CLIA and BDG CLIA.

			Platelia GM ELISA	Dynamiker GM ELISA	GM CLIA	BDG CLIA
Spearman's rho	Platelia GM ELISA	Correlation coefficient	1000	0.618^a^	0.352^a^	0.446^a^
		Sig. (2‐tailed)		0.000	0.001	0.000
		*N*	93	93	93	93
	Dynamiker GM ELISA	Correlation coefficient	0.618^a^	1000	0.364^a^	0.483^a^
		Sig. (2‐tailed)	0.000		0.000	0.000
		*N*	93	93	93	93
	GM CLIA	Correlation coefficient	0.352^a^	0.364^a^	1000	0.414^a^
		Sig. (2‐tailed)	0.001	0.000		0.000
		*N*	93	93	93	93
	BDG CLIA	Correlation coefficient	0.446^a^	0.483^a^	0.414^a^	1000
		Sig. (2‐tailed)	0.000	0.000	0.000	
		*N*	93	93	93	93

*Note:* The correlation coefficients (*ρ*) indicate the strength and direction of monotonic relationships between the variables. Statistically significant correlations at the 0.01 level (two‐tailed) are marked with (*p* < 0.01). *N* = 93 for all comparisons.

^a^ Correlation is significant at the 0.01 level (2‐tailed).

## 4 Discussion

Delayed diagnosis of IA can lead to late initiation of treatment, increasing morbidity and mortality among immunocompromised patients. The low positivity rates of tissue or blood culture necessitate alternative non‐culture‐based diagnostic methods. In this context, serological tests emerge as rapid and noninvasive alternatives for IA diagnosis. Galactomannan (GM) testing is among the most commonly used antigens for diagnosing IA. GM, produced by *Aspergillus* species, is a biomarker frequently detected in neutropenic and immunocompromised patients [13].

In this study, the FDA‐approved Platelia GM ELISA test served as the reference standard, and other tests were compared against it. DynGM ELISA demonstrated high concordance with Platelia GM ELISA, showing superior sensitivity and specificity, particularly for diagnosing IA. While GM tests in the literature showed sensitivity ranges of 70%–85% [14, 15], in our study, DynGM ELISA exhibited a sensitivity of 100%, specificity of 97.4%, PPV of 89.5% and NPV of 100%. The higher sensitivity observed in our study may be attributed to the fact that the high‐probability IA group consisted of patients with haematological malignancies. Two additional GM‐negative samples were found to be falsepositives by DynGM ELISA. These two patients had no IFIs; one was AML, and the second one was a multiple myeloma patient. In some reports, multiple myeloma was considered a major cause of false‐positive galactomannan tests because of the underlying paraproteinemia [16].

Fernandez‐Cruz et al. showed 85% specificity for DynGM ELISA tested bronchoalveolar lavage fluid, highlighting its utility in neutropenic patients [17]. Eigl et al.'s multicenter evaluation noted high sensitivity in ICU patients but variable specificity [18]. In a meta‐analysis involving immunocompromised and neutropenic patients, when the OD index for GM ELISA was set at 0.5, the sensitivity was found to be 78% (70%–85%), and specificity was 85% (78%–91%). When the OD index was set at 1.0, the sensitivity decreased to 71% (63%–78%) while the specificity increased to 90% (86%–93%) [19]. As the optical density threshold increased, sensitivity decreased while specificity improved. In our study, the optical density cut‐off value was set at 0.5 for both ELISA tests.

In a study conducted by Çuhadar et al. the diagnostic sensitivity of the DynGM ELISA and Dynamiker BDG tests for invasive pulmonary aspergillosis (IPA) infections were compared to the FDA‐approved Platelia GM ELISA test. The study included a total of 62 patients, 32 (51.6%) of whom were diagnosed with IPA, while the remaining 30 (48.4%) patients formed the control group. Among the 32 IPA‐diagnosed patients, 11 (34.4%) were classified as having probable IPA and 21 (65.6%) as having possible IPA. When the GM ELISA was taken as the reference, the DynGM ELISA test demonstrated a sensitivity of 77.8%, specificity of 93.7%, PPV of 63.6%, NPV of 96.7% and accuracy of 91.7% (*p* < 0.001) [20].

In a retrospective study conducted on serum samples from 81 patients with haematological malignancies (28 probable, 23 possible IPA and 30 negative cases), the DynGM ELISA demonstrated a sensitivity of 79.3%, specificity of 83%, PPV of 71.9% and NPV of 88% [21]. In a study conducted by Guo et al. involving 228 patient serum samples and using the EORTC/MSG criteria as the reference, the DynGM ELISA demonstrated a sensitivity of 80.73%, specificity of 97.48%, PPV of 96.70% and NPV of 84.67%. The area under the ROC curve (AUC) was calculated as 0.901 [22]. In our study, consistent with these findings, the AUC value for the DynGM ELISA was found to be 0.994.

Chemiluminescence immunoassay (CLIA) methods have gained attraction in recent years for their rapid results and automation. Literature reports CLIA GM sensitivities ranging from 60% to 85%, aligning with the study's findings [11, 12]. The superior sensitivity of ELISA suggests that it remains a more reliable method for multi‐sample testing. CLIA's main advantage over ELISA is faster results in a closed system, reducing contamination risk and manual workload. However, ELISA's higher sensitivity positions it as a preferred option for diagnostic reliability. In our study, when the Platelia ELISA was taken as the reference, the GM CLIA test demonstrated a sensitivity of 70.6% and a specificity of 92.1%. GM CLIA testing provides a practical option for quick hospital diagnostics but showed lower sensitivity compared to GM ELISA in our study. The GM CLIA test demonstrated a PPV of 66.7% and an NPV of 93.3%. According to the AUC value of the ROC curve, the GM CLIA test was found to be 86.9% accurate.

In a study conducted by Troncoso et al. the Platelia *Aspergillus* Ag test was compared with the *Aspergillus* GM CLIA test. The two methods showed an agreement of 85.7% in serum samples. The GM CLIA test demonstrated approximately 16% higher sensitivity compared to GM ELISA, while the GM ELISA exhibited 11% greater specificity than the GM CLIA [23]. In an unpublished study conducted by Wang et al. 126 IA cases (43 proven, 83 probable) and 195 negative serum samples were collected. The diagnosis of IA was made according to the EORTC/MSG guidelines. A total of 326 serum samples were tested using both the Platelia *Aspergillus* Ag test and GM CLIA test. Compared with the GM ELISA test, the GM CLIA test demonstrated a sensitivity of 83.33% and specificity of 82.05% [24]. In our study, the lower sensitivity (70.6%) of GM CLIA was attributed to the smaller number of patients diagnosed with IA.

In a study conducted by Kupper et al. the performance of the GM CLIA test was evaluated in a group of 101 patients who had undergone allogeneic HSCT, including four patients with proven or probable IA. For a single serum sample, the GM CLIA test demonstrated a sensitivity of 75.0%, specificity of 88.7%, PPV of 21.7% and NPV of 98.8%. The ROC analysis yielded an AUC value of 0.819 [25].

Calero et al. compared two different *Aspergillus* GM antigen tests. Samples from 327 patients were tested using the GM CLIA test and the Platelia *Aspergillus* Ag ELISA test. Among these, 120 samples were analysed retrospectively (95 proven/probable IA cases and 25 healthy controls), and 207 samples were analysed prospectively. Accepting the Platelia *Aspergillus* Ag ELISA test as the reference, the GM CLIA test demonstrated a sensitivity of 93.8% and a specificity of 89.5%, with the area under the ROC curve (AUC) calculated as 0.97. When only the retrospective group was evaluated using the EORTC/MSG criteria as the reference, the sensitivity, specificity, PPV and NPV of the GM ELISA test were found to be 63.2%, 100%, 100% and 41.7%, respectively. For the GM CLIA test, these values were 80.9%, 100%, 100% and 59.5%, respectively. The AUC values for the ROC curve were calculated as 0.962 for the GM ELISA and 0.968 for the GM CLIA test [26].

In a study by Albert et al. the GM ELISA and GM CLIA tests were compared using serum and lower respiratory tract samples. A total of 535 samples (320 serum, 86 bronchial aspirates, 70 BAL and 59 ETA) from 177 adult patients were tested using both methods. The patient groups included one proven IPA case, 11 probable IPA cases and 33 possible IPA cases. For proven/probable IPA patients, the overall sensitivity and specificity of the GM CLIA test were found to be 100% and 65%, respectively. For serum samples, the PPV and NPV were calculated as 42% and 92%, respectively. ROC analysis performed exclusively on serum samples yielded AUC values of 0.80 for the GM CLIA test and 0.87 for GM ELISA [27].

In a recent retrospective study conducted by Schub et al. five *Aspergillus* antigen assays, including GM CLIA test and one BDG assay, were compared. Blood samples of 82 patients (81 sera and one plasma sample) with proven (*n* = 11) or probable (*n* = 71) IA according to the revised and updated consensus definitions of invasive fungal disease of the EORTC/MSG definitions were collected. The study revealed analytic data for GM CLIA test, having an accuracy of 0.72%–0.76% (depending on estimated prevalence rates of 1%, 5% and 10%), sensitivity of 34%, specificity of 76% and NPV 99.14% in the haematological malignancy group of patients [11].

Buil et al. [12] assessed the VirClia Galactomannan (GM) assay for detecting IPA using BAL fluid from haematological patients. The study compared the GM CLIA method with the traditional Platelia GM ELISA across 141 samples. Results showed comparable sensitivity and specificity for the two assays, with strong quantitative correlation (Spearman's rho = 0.72). The CLIA offered faster results due to its non‐batching set‐up, making it advantageous in clinical scenarios requiring quick diagnostics. However, both assays showed moderate sensitivity (ranging from 57% to 76%) and high specificity. The performance of CLIA was consistent across various cut‐off thresholds, and discrepancies between CLIA and ELISA results were primarily around these thresholds. While both tests were effective in distinguishing IPA from controls, the study recommended combining multiple diagnostic approaches, including culture and PCR, for comprehensive IPA diagnosis. The findings highlight CLIA as a viable alternative to ELISA, particularly in settings prioritising speed and efficiency. Overall, our GM CLIA results were found to be comparable to the related literature with 70.6% sensitivity. The PPV of GM CLIA, on the other hand, was generally found to be limited in the literature, requiring confirmatory ELISA tests to clarify real positivity. Our results and similar articles indicated that certain tests should only be used in certain situations: For screening purposes in high‐risk patients, there is a particular need for a low false‐positivity rate like we see in ELISAs due to their high specificity. However, cost‐effectiveness should be considered. In differential diagnosis in case of suspicion of fungal infection, tests with a higher sensitivity, like CLIA assays, could demonstrate their strength [28].

In addition to the increasing number of GM assays validated for clinical use, it is important to highlight emerging alternatives beyond the established Dynamiker and Bio‐Rad assays. Notably, the Euroimmun *Aspergillus* Antigen ELISA has demonstrated strong diagnostic performance, with a sensitivity of 90% and specificity of 96% at an optimised cut‐off, and an area under the ROC curve (AUC) of 0.959, closely matching the performance of the Platelia GM ELISA in BAL fluid [29]. Similarly, the IMMY *Aspergillus* GM ELISA, particularly the Clarus prototype, showed excellent concordance with Platelia GM, achieving a sensitivity of 96% and specificity of 74%–81% depending on the cut‐off used, with an AUC of 0.936. Furthermore, lateral flow assays (LFAs) are increasingly recognised for their rapidity and practicality in clinical settings [30]. The IMMY LFA, in particular, demonstrated comparable or superior sensitivity to ELISA in BAL samples and showed promising utility in diagnosing COVID‐19‐associated pulmonary aspergillosis (CAPA) and influenza‐associated pulmonary aspergillosis (IAPA) [31]. Due to the non‐specific clinical and radiological features of CAPA, mycological evidence plays a central role in diagnosis. Traditional methods such as culture suffer from low sensitivity and delayed results, making non‐culture‐based methods highly valuable. In one study, GM detection in BAL and serum using both EIA and LFA demonstrated moderate to high sensitivity (BAL LFA: 60.6%, BAL EIA: 54.5%) and excellent specificity (BAL LFA: 88.9%, BAL EIA: 91.7%), with LFA showing a strong correlation with EIA and better accessibility in low‐resource settings [32]. Another study explored the use of GM detection in tracheal aspirate (TA) samples using the IMMY Sona *Aspergillus* LFA, revealing high diagnostic accuracy with 93% sensitivity and 93% specificity, and almost perfect agreement with EIA (Cohen's kappa = 0.83) [33]. These findings emphasise the need to consider a broader spectrum of validated GM assays, including novel ELISAs and LFAs, in both research and clinical practice.

Beta‐D‐glucan (BDG) testing, a pan‐fungal biomarker, detects components in the cell walls of species such as *Candida*, *Aspergillus* and *Pneumocystis*. While useful for broad‐spectrum fungal screening, its lack of pathogen specificity is a significant limitation. BDG serves as a sensitive screening tool for all fungal infections, while GM provides specificity for *Aspergillus* infections [34]. In our study, based on the Platelia GM ELISA test results for a single serum sample, the BDG CLIA test demonstrated a sensitivity of 88.2%, specificity of 81.6%, PPV of 51.7% and NPV of 96.9%. The ROC analysis resulted in an AUC value of 0.878.

In the literature, BDG and GM results were compared in terms of diagnostic performance for IA. However, the majority of BDG testing is based on limulus lysate assay [35, 36]. There are limited data about BDG CLIA tests. In a study, 52 samples were obtained from proven IPA patients, 140 samples from probable IPA patients, and 382 samples from non‐IPA patients or healthy volunteers. The samples were tested simultaneously for BDG antigen detection using both CLIA and ELISA methods. ROC analysis was performed for both tests, with the area under the curve (AUC) calculated as 0.905. For the BDG antigen test analysed by the CLIA method, sensitivity and specificity were calculated as 83.33% and 89.76%, respectively [37]. In another study, aimed to assess the diagnostic performance of the same methodology that we performed, FungiXpert Fungus BDG Detection Kit by Genobio Pharmaceutical Co. Ltd. (Tianjin, China) was used. They evaluated the same chemiluminescent method for the diagnosis of candidemia and deep‐seated candidiasis [38]. They found the sensitivity, specificity, positive and negative predictive values were 60.52%, 81.81%, 85.18% and 54.54%, respectively. The positive likelihood ratio was 3.32. Similar to our results, they concluded that the performance of the BDG CLIA test was acceptable for invasive candidiasis in the present resource‐limited set‐up. They noticed the major advantages of this assay, as we noticed, the ease of performance in a semi‐automated format, relatively lower cost per test, non‐reliance on glucan‐free procedures or instruments and minimal hands‐on procedures.

Our study's limited sample size necessitates further validation in larger populations. Evaluating test sensitivity and specificity in non‐haematologic malignancy groups could provide a broader perspective. Additionally, exploring GM and BDG tests' applicability in noninvasive samples, such as urine or saliva, is crucial.

Future research should optimise these tests' diagnostic performance and evaluate new‐generation serological tests' contributions to improving clinical decision‐making and patient survival rates.

## 5 Conclusion

The critical role of early diagnosis in managing IFIs underscores the importance of serological testing. Dynamiker GM's high sensitivity and specificity position it as a reliable biomarker, while CLIA‐based methods offer rapid results valuable for clinical practice. Combining BDG and GM tests enhances diagnostic accuracy and minimises false positives. This study contributes to understanding CLIA tests' clinical utility and highlights the need for broader studies to validate these findings and improve clinical reliability. High NPV of BDG CLIA test revealed its utility for screening high‐risk patients. However, further studies involving larger patient groups are necessary to validate these findings. Early diagnosis enables timely initiation of appropriate treatment, which is beneficial in reducing mortality.

## Author Contributions

**Esra Kılıç:** data curation, investigation, methodology. **Elif Ayça Şahin:** investigation, visualization, formal analysis, supervision, data curation, methodology. **Özlem Güzel Tunçcan:** investigation, visualization, data curation, methodology. **Şeyma Yıldız:** investigation, data curation, methodology. **Zübeyde Nur Özkurt:** data curation, methodology, formal analysis. **Zeynep Arzu Yeğin:** data curation, writing – review and editing, methodology, formal analysis. **Ayşe Kalkancı:** funding acquisition, writing – original draft, investigation, validation, project administration, supervision, data curation, conceptualization, visualization, resources.

## Data Availability

The data that support the findings of this study are available on request from the corresponding author. The data are not publicly available due to privacy or ethical restrictions.
